# Rapid genotyping protocol to improve dengue virus serotype 2 survey in Lao PDR

**DOI:** 10.1371/journal.pone.0237384

**Published:** 2020-08-07

**Authors:** Elodie Calvez, Somphavanh Somlor, Souksakhone Viengphouthong, Charlotte Balière, Phaithong Bounmany, Sitsana Keosenhom, Valérie Caro, Marc Grandadam

**Affiliations:** 1 Institut Pasteur du Laos, Vientiane, Lao People’s Democratic Republic; 2 Institut Pasteur, Paris, France; 3 Institut de Recherche Biomédicale des Armées, Brétigny-sur-Orge, France; CEA, FRANCE

## Abstract

Dengue fever is one of the major public health problems in Lao PDR. Over the last decade, dengue virus (DENV) epidemics were characterized by a novel predominant serotype accompanied by at least two other serotypes. Since 2008, DENV-2 circulated at a low level in Lao PDR but its epidemiologic profile changed at the end of 2018. Indeed, the number of confirmed DENV-2 cases suddenly increased in October 2018 and DENV-2 became predominant at the country level in early 2019. We developed a Genotype Screening Protocol (GSP) to determine the origin(s) of the Lao DENV-2 and study their genetic polymorphism. With a good correlation with full envelope gene sequencing data, this molecular epidemiology tool evidence the co-circulation of two highly polymorphic DENV-2 genotypes, i.e. Asian I and Cosmopolitan genotypes, over the last five years, suggesting multiple introductions of DENV-2 in the country. GSP approach provides relevant first line information that may help countries with limited laboratory resources to reinforce their capabilities to DENV-2 and to follow the epidemics progresses and assess situations at the regional level.

## Introduction

Dengue fever is the most prevalent arboviral disease in the world. It represents a threat for 390 million people in 128 countries primarily in Latin America, Western Pacific and in South East Asia [1, 2]. The World Health Organization (WHO) estimated that at least 500,000 people, mostly children, are hospitalized annually; of whom 2.5% have fatal outcomes.

Lao People's Democratic Republic (PDR) is a low-middle income country located at a central position of the Indochinese peninsula. Lao PDR has a population of 6.76 million with nearly 40% under the age of 20 (sources: WHO; www.lsb.gov.la). The first dengue virus (DENV) outbreak in Lao PDR was reported in 1979 [3]. Since then, several epidemics have been recorded in the country that raise dengue to the rank of a major public health concern for their national authorities [3]. Previous studies have highlighted the dramatic increase in commercial and tourism exchanges between Lao PDR and its neighboring countries as the driving force behind the entry of multiple DENV serotypes and/or genotypes into the country [4, 5]. The emergence of a new DENV serotype or genotype has been reported to enhance the number of cases and the diseases’s severity [6–8].

**Fig 1:**
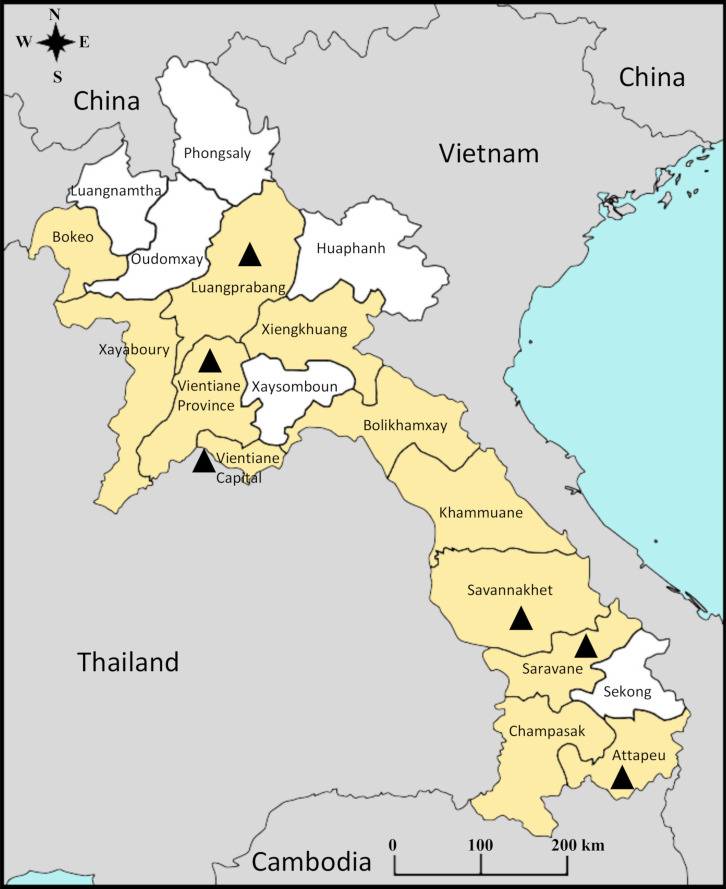
Map of Lao PDR. Provinces with DENV-2 reported cases are in yellow. Triangles indicate the localization of DENV-2 isolates which were sequenced in this study. This map was generated by IPL staff using Inkscape software free of copyright.

In 2012, an arbovirus laboratory surveillance system was set up in the capital city of Vientiane to improve arboviral diagnosis and prevention in the country [5, 9, 10]. Since 2012, the Vientiane Capital Province has experienced two major DENV epidemics, first with a predominance of DENV-3 in 2012–2013 and then DENV-4 from the end of 2014 to end of 2018 [4, 5, 9, 11]. Over this period, DENV-2 was only the cause of 3 to 20% of confirmed cases [5] (https://www.pasteur.la/). However, in October 2018 DENV-2 burden progressively increased and reached 43% of the confirmed dengue infections in Vientiane capital the following December [12]. Since the beginning of 2019, DENV-2 has become the predominant serotype; representing 68.9% of the samples serotyped in the capital [13]. Of the 21 fatal cases recorded in 2019 in Vientiane Capital by the arbovirus surveillance system, six could be serotyped, of which four were assigned to DENV-2. At the country level, DENV-2 has been reported in 12 of the 18 provinces (Fig 1).

Little is known concerning DENV-2 genotype circulation and phylogeny in Lao PDR. Here we report on the development of a Genotype Screening Protocol (GSP) based on partial E gene sequencing for the rapid identification of DENV-2 genotyping and its origin. Performance of the GSP was compared to full E gene sequencing. GSP was also retrospectively applied to a panel of DENV-2 positive samples or isolates collected over the last eight years in Vientiane Capital as well as other Lao provinces.

## Materials and methods

### Sample collection

A panel of 53 DENV-2 isolates, screened by the arbovirus surveillance network as previously described [5], of autochthonous (51 samples) and imported cases (2 samples) across six Lao provinces between 2012 to 2019, was analyzed (Fig 1; Table 1).

**Table 1:** References of Lao DENV-2 isolates.

Sample identification	Years of collection	RNA source	GenBank accession number		Cq^(^^2^^)^
			GSP^(^^1^^)^	Full Envelopee protein	
LaoPDR-Vientiane Capital -2015-3231	2015	Culture supernatant	MN444556	MN444605	27
LaoPDR-Vientiane Capital -2016-3550	2016	Culture supernatant	MN444557	MN444606	24
LaoPDR-Vientiane Capital -2016-3771	2016	Culture supernatant	MN444558	MN444607	26
LaoPDR-Vientiane Capital -2016-3831	2016	Plasma	MN444559	MN444608	25
LaoPDR-Vientiane Capital -2017-5207	2017	Culture supernatant	MN444560	MN444609	20
LaoPDR-Vientiane Capital -2017-5208	2017	Culture supernatant	MN444561	MN444610	21
LaoPDR-Vientiane Capital -2017-5443	2017	Culture supernatant	MN444562	MN444611	24
LaoPDR-Vientiane Capital -2017-5902	2017	Plasma	MN444563	F	20
LaoPDR-Vientiane Capital -2017-5955	2017	Culture supernatant	MN444564	F	25
LaoPDR-Vientiane Capital -2017-6086	2017	Plasma	MN444565	F	21
LaoPDR-Vientiane Capital -2017-6177	2017	Plasma	MN444566	F	25
LaoPDR-Vientiane Capital -2017-6446	2017	Culture supernatant	MN444567	MN444612	22
LaoPDR-Vientiane Capital -2018-7406	2018	Culture supernatant	MN444568	MN444613	22
LaoPDR-Vientiane Capital -2018-7692	2018	Plasma	MN444569	ND	31
LaoPDR-Vientiane Capital -2018-7740	2018	Plasma	MN444570	F	22
LaoPDR-Vientiane Capital -2018-7800	2018	Plasma	MN444571	ND	23
LaoPDR-Vientiane Capital -2018-7944	2018	Plasma	MN444572	F	23
LaoPDR-Vientiane Capital -2018-8075	2018	Plasma	MN444573	ND	21
LaoPDR-Vientiane Capital -2018-8173	2018	Plasma	MN444574	MN444614	22
LaoPDR-Vientiane Capital -2018-8359	2018	Plasma	MN444575	ND	25
LaoPDR-Vientiane Capital -2018-8366	2018	Plasma	MN444576	ND	26
LaoPDR-Vientiane Capital (fatal case)-2018-8372	2018	Culture supernatant	MN444577	MN444615	18
LaoPDR-Vientiane Capital -2018-8509	2018	Plasma	MN444578	ND	29
LaoPDR-Vientiane Capital -2018-8518	2018	Plasma	MN444579	ND	24
LaoPDR-Vientiane Capital -2018-8541	2018	Plasma	MN444580	ND	31
LaoPDR-Vientiane Capital (fatal case)-2018-8920	2018	Plasma	MN444581	MN444616	23
LaoPDR-Vientiane Capital (fatal case)-2019-9060	2019	Plasma	MN444582	F	36
LaoPDR-Vientiane Capital (fatal case)-2019-9080	2019	Culture supernatant	MN444583	MN444617	18
LaoPDR-Vientiane Capital -2019-9128	2019	Plasma	MN444584	ND	20
LaoPDR-Vientiane Province-2017-5545	2017	Plasma	MN444585	F	29
LaoPDR-Vientiane Province-2017-5725	2017	Plasma	MN444586	F	25
LaoPDR-Vientiane Province-2017-5942	2017	Culture supernatant	MN444587	ND	24
LaoPDR-Luangprabang-2017-5705	2017	Plasma	MN444588	MN444618	24
LaoPDR-Luangprabang-2018-8737	2018	Plasma	MN444589	F	26
LaoPDR-Luangprabang-2018-8741	2018	Plasma	MN444590	F	25
LaoPDR-Savannakhet-2018-8014	2018	Plasma	MN444591	MN444619	25
LaoPDR-Savannakhet-2018-8135	2018	Plasma	MN444592	ND	20
LaoPDR-Savannakhet-2018-8358	2018	Plasma	MN444593	F	29
LaoPDR-Saravane-2018-7670	2018	Plasma	MN444594	F	26
LaoPDR-Saravane-2018-7936	2018	Plasma	MN444595	F	21
LaoPDR-Saravane-2019-9444	2019	Plasma	MN444596	ND	25
LaoPDR-Saravane-2019-9474	2019	Plasma	MN444597	ND	21
LaoPDR-Attapeu-2017-5601	2017	Culture supernatant	MN444598	MN444620	23
LaoPDR-Attapeu-2017-6144	2017	Plasma	MN444599	F	31
LaoPDR-Attapeu-2018-7507	2018	Plasma	MN444600	F	23
LaoPDR-Attapeu-2018-7511	2018	Plasma	MN444601	MN444621	24
LaoPDR-Attapeu-2019-9494	2019	Plasma	MN444602	ND	22
**LaoPDR-Vientiane Capital (ex Thailand)-2017-5642**	**2017**	**Plasma**	**MN444603**	**MN444622**	**27**
**LaoPDR-Vientiane Capital (ex Vietnam)-2018-8746**	**2018**	**Plasma**	**MN444604**	**F**	**30**

^(1)^ Genotype Screening Protocol.

^(2)^ Cq were obtained from plasma or culture supernatant with pan-dengue RT-PCR from Warrilow *et al* [14]. ND: not done. F: Failed. Imported cases are indicated in bold.

### Ethic statement

The National Ethic Committee for Health Research of the Ministry of Health of Lao PDR approved the protocol used in this study. This protocol was also approved by all public hospitals' management committees and the agreement of the Ministry of health was obtained.

For all the patient samples included in this study, a written informed consent was provided by all the adult volunteers or by a parent or legal guardian on behalf of their children.

### Screening of dengue suspected cases and serotype identification

RNA was extracted from the plasma of suspected dengue cases using Nucleospin RNA virus kit (Macherey-Nagel) following the manufacturer’s instructions. Sample RNA was first screened by mean of a pan-dengue virus one-step RT-PCR as described previously [5, 14]. DENV serotype identification was tentatively determined using primer/probe sets described by Ito *et al* adapted to a multiplex one-step RT-PCR format [15].

### Gene E sequencing analysis

The DENV genotypes and their geographic origin(s) were initially determined by the rapid Genotype Screening Protocol (GSP) based on partial envelope gene sequencing. Viral genome RNA was extracted from human plasma when the Cq value of the screening RT-PCR was below 30. For samples which displayed a Cq value above 30, and when the remaining volume was sufficient, DENV was isolated on C6/36 monolayers to improve the chance of sequencing. Such additional viral amplification has been documented to have no impact on viral genome stability [16]. In these cases, the above RNA extraction protocol was applied to viral culture supernatants. For sequencing purposes, first strand cDNA was generated using random hexamers and the Maxima H Minus First Strand cDNA Synthesis kit (Thermo Scientific). PCR was performed using Phusion Flash High-Fidelity PCR Master Kit (New England Biolabs^®^ Inc). GSP (552 nt) was performed using FG4 primers (Table 2). Complete envelope gene sequences (1485 nt) were established for a panel of samples (Table 2) selected within the clusters obtained by GSP using primers FR2 or FGT2 (respectively adapted for Cosmopolitan and Asian I genotypes), FGT3 and FG4 (Table 2). Amplified fragments were purified using ExoSAP-IT^TM^ PCR Product Cleanup Reagent (Thermo Fisher Scientific) or by purification of PCR products from agarose gel using the Cleanup Gel extraction kit (Macherey-Nagel) when nonspecific amplification was observed by agarose gel electrophoresis. Forward and reverse strands were independently sequenced using BigDye Terminator v3.1 Cycle sequencing kit (Applied Biosystem) and loaded on a Genetic Analyzer 3500xL (Applied Biosystem).

**Table 2:** List and positions of primers used for partial or complete envelope gene RT-PCR and sequencing.

Fragment	Forward	Genome position	Reverse	Genome position
FR2^a^	^5’^TGT-CAT-CAG-AAG-GGG-CCT-G^3’^	770–788	^5’^TCA-TTG-AAG-TCR-AGG-CCC-G^3’^	1502–1520
FGT2 ^a^	^5’^TCA-CCA-TAA-TGG-CAG-CAA-TC^3’^	836–855	^5’^TGC-ACC-AGC-CAA-GCT-TTA-TT^3’^	1543–1562
FGT3 ^a^	^5’^ACA-CCA-TTG-TGR-TAA-CAC-C^3’^	1346–1364	^5’^TCT-GCT-TCT-ATG-TTG-ACT-G^3’^	2027–2045
FG4 ^a^^,^ ^b^	^5’^TGT-GAA-GGA-AAT-AGC- AGA ^3’^	1860–1877	^5’^AGT-TCT-TTG-TTT-TTC-CAG-CT ^3’^	2440–2459

The genome positions are given according to the dengue virus serotype 2 reference genome (GenBank: U87411).

^a^ indicates the primers used for complete envelope gene sequencing and

^b^ for the Genotype Screening Protocol.

### Phylogenetic analysis

Partial (53 sequences; GenBank MN444556 to MN444604) and complete (22 sequences; GenBank MN444605 to MN444622) envelope sequences of Lao isolates (Table 1) were compared with envelope gene sequences retrieved from GenBank (S1 Table) [4, 17–44]. Maximum-likelihood trees were constructed using MEGA version 7 (www.megasoftware.net) as previously described [5, 6, 45–47], with the Kimura-2-parameter method and a bootstrap of 1000 replications [48].

## Results

### Co-circulation of two DENV-2 genotypes in Lao PDR

**Fig 2:**
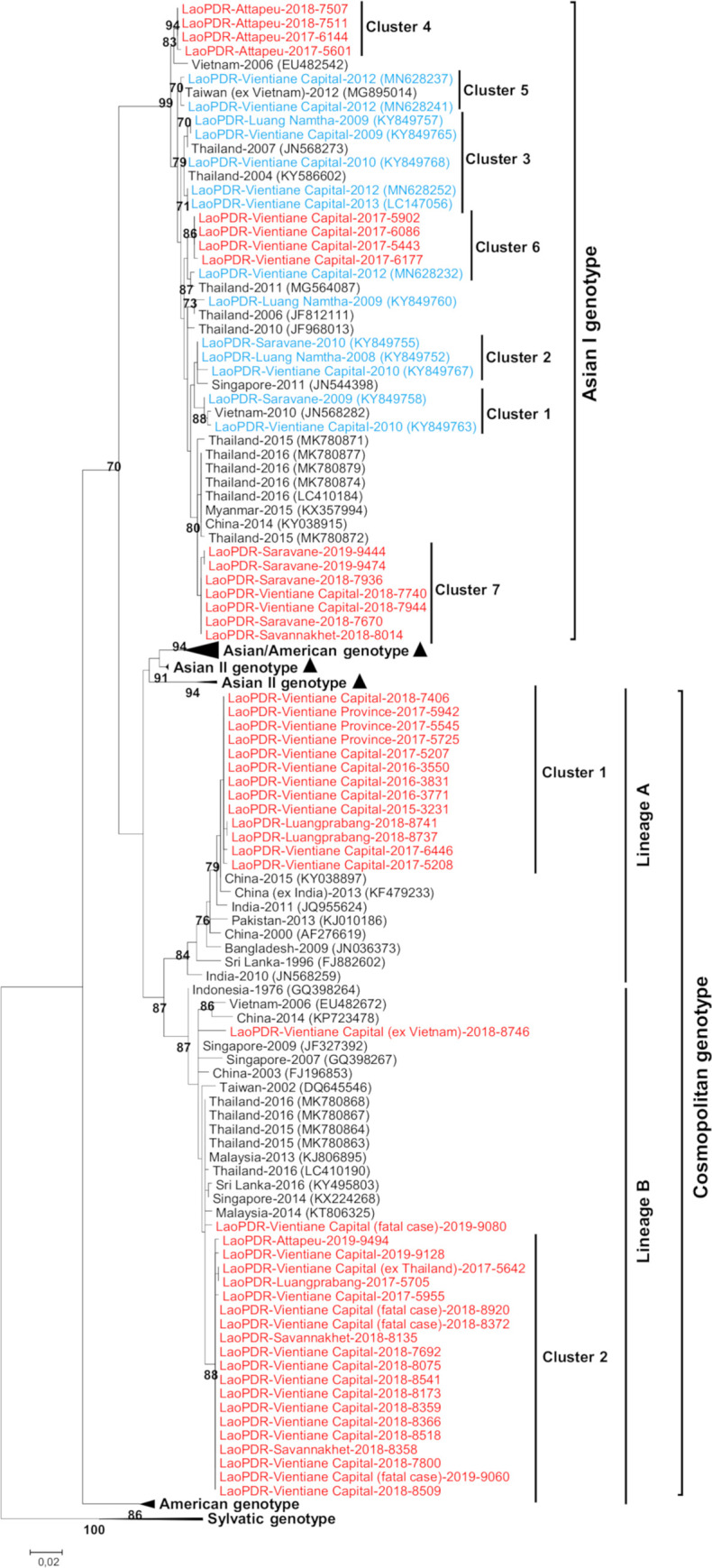
Maximum-likelihood phylogenetic tree of DENV-2 sequences from Lao PDR. The tree was constructed on a 552nt segment of a partial envelope protein gene (Genotype Screening Protocol). Bootstrap values >70 are shown next the node. The Lao PDR strains analyzed in this study are indicated in red. The Lao PDR strains previously described are in blue. Cluster 1–3 were previously described by Castonguay-Vanier *et al*). Scale bar indicates nucleotide substitution per site. The triangles indicate discordance between Genotype Screening Protocol and full envelope protein gene sequencing at the genotype and lineage level.

Of this panel of 53 isolates, GSP verified the co-circulation of two DENV-2 genotypes. Asian I and Cosmopolitan genotypes were both detected in different Lao PDR provinces (Fig 2). The co-circulation of these genotypes was detected in 2017, 2018 and 2019 in the country.

### Persistence of DENV-2 Asian I genotype in Lao PDR

Lao DENV-2 samples isolated between 2008 and 2010 have been assigned to the Asian I genotype [4]. In our series, 19 isolates (36%) obtained after 2012 shared strong identity (>97.2%) with this genotype and demonstrates its persistence in the country for over eleven years. Lao Asian I isolates since 2008 were classed into seven clusters, displaying distances of 1.0 to 2.8% from each other at the nucleotide level (Fig 2). Three of these clusters were described previously and one was found specifically from the Attapeu province [4]. All the Lao isolates showed strong links with strains from Vietnam, Thailand, China, Taiwan and Myanmar.

### Co-circulation of different lineages and clusters within DENV-2 cosmopolitan genotype

A total of 34 isolates (64%) belonged to the Cosmopolitan genotype, and analysis showed that it may has been circulating in Lao PDR since at least 2015. Interestingly, the Cosmopolitan Lao isolates split into two distinct lineages (A; B), displaying 4.9–5.7% divergence from each other (bootstrap 87%). These lineages have coexisted at least as early as 2017. Within Lineage A, all Lao isolates grouped together sharing more than 99.45% identity (Cluster 1). Strong links (>98.7% identity; bootstrap 79%) were found between Lineage A with DENV-2 strains circulating in China and India in 2015 and 2011/2013 respectively. All four fatal cases included in this series belonged to Lineage B (Fig 2). Three of the four fatal cases grouped with the rest of the autochthonous isolates in a major cluster (Cluster 2, Fig 2). Surprisingly, the last fatal case (LaoPDR-Vientiane Capital-2019-9080) displayed between 1.1 and 2.1% nucleotide distance with the rest of Cluster 2. Autochthonous Lao isolates belonging to Lineage B strongly linked with DENV-2 strains from Thailand/Malaysia/Singapore/Sri-Lanka. One of the two imported cases (ex Thailand), fell in Cluster 2 whereas the second (ex Vietnam) grouped with other regional isolates detected in China, India, Bangladesh, Sri Lanka, and Southeast Asia.

### Validation of a Genotype Screening Protocol

**Fig 3:**
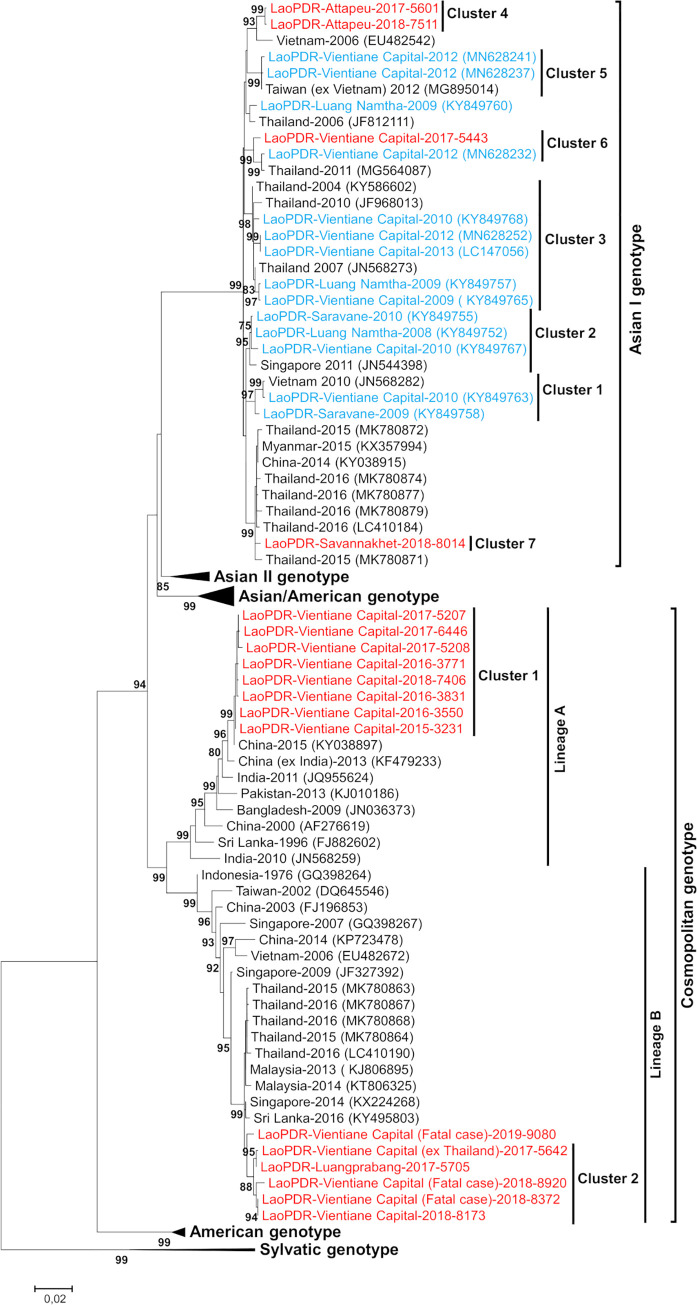
Maximum-likelihood phylogenetic tree of DENV-2 sequences from Lao PDR. The tree was constructed on a 1485nt segment of the envelope protein gene. Only the bootstrap values >70 are shown. Scale bar indicates nucleotide substitution per site. The Lao PDR strains analyzed in this study are indicated in red. The Lao PDR strains previously described are in blue. Cluster 1–3 were previously described by Castonguay-Vanier *et al*).

To assess the accuracy of the GSP, the genotype/lineage/cluster assignments of 8 Asian I and 14 Cosmopolitan isolates were checked by full length envelope gene sequencing (Fig 3). The distribution of reference strains and Lao isolates established by the full envelope genotyping globally matched with the GSP results including at the levels of lineages and/or clusters. However, differences in the topology of the trees were observed for the Asian II genotype, for which some strains were previously found more closely related to the Asian/American genotype by GSP. At a higher level, the Asian II and the Asian/American genotypes were linked to the Cosmopolitan genotype by GSP rather than to the Asian I genotype as with full envelope sequencing (Fig 3).

In the subset selected for the complete envelope gene sequencing, sequences could not be established for some samples (plasma or culture supernatants) with a Cq>30 when tested with the screening real time RT-PCR (Table 1). For instance, from the 4 investigated fatal DENV-2 cases, the genotype of one sample (LaoPDR-Vientiane Capital-2019-9060) with a Cq = 36 could only be determined by GSP whereas the full envelope gene sequencing failed.

## Discussion

Dengue molecular epidemiology in Lao PDR is complex and the dynamic over time depends in part on virus serotypes and geographic origins [4, 5]. Indeed, Lao PDR is localized in a hyperendemic DENV region in Southeast Asia in the middle of the Indochinese peninsula [49]. Since the first dengue outbreak in 1979, the four DENV serotypes are frequently detected in the country [3–5, 9, 11]. DENV genotype co-circulation has already been depicted in Lao PDR for DENV-3 [5]. For DENV-2, the co-circulation of Asian I and Asian/American or Cosmopolitan genotypes has been documented in Thailand, Cambodia or Vietnam [43, 50].

In most of these situations, the genotypes coexisted over several years [50]. In Lao PDR, Asian I and Cosmopolitan genotypes have been co-circulating since at least 2017 and was still ongoing in 2019. Compared to a recent study in Thailand, our series identified different lineages within the DENV-2 Cosmopolitan genotype [43]. Indeed, in Thailand only the Cosmopolitan genotype/Lineage B was observed between 2015 and 2016. As seen elsewhere, since 2008 the Lao clusters within the Asian I genotype have had different geographic origins and evolved over time as independent topotypes as is seen on other countries [4, 43, 50]. In a panel tested previously and in this study, the co-circulation of two or three clusters were observed in 2009–2010 (cluster 1, 2 and 3), in 2012 (clusters 3 and 5), in 2017 (clusters 4 and 6) and in 2018 (clusters 4 and 7). Interestingly, some clusters seemed to be localized only in some part of Lao PDR such as clusters 2 and 6 in Vientiane, and cluster 4 in Attapeu. Though, this observation could be due to the limited size of the series. However, the close relation between cluster 4 isolates and Vietnamese prototype strains supports another hypothesis. It can be assumed that this cluster originates from a direct introduction from Vietnam as the Eastern limit of the province materialize part of the Lao-Vietnam border. A main road, rubber plantations held by Vietnamese companies, are factors that may at least in part facilitate the traffic of DENV virus in southern Lao PDR. Further investigation is needed to better document the cluster-specific circulation of DENV-2 Asian I genotype in Lao PDR and to determine factors which could impact the selection of specific DENV strains. For instance, it has already been demonstrated that specific adaptations between the vector population and DENV may lead to an enhancement of mosquitos’ ability to transmit DENV [51, 52].

GSP results showed independent introduction events of DENV-2 between 2012 and 2019 in Lao PDR. Genetic links and the geographic origin of the imported cases suggest a possible role of human regional mobility as a factor of DENV-2 polymorphism through the introduction of novel genotype, lineage, or clusters into the country. Observations of the presence of the Cosmopolitan genotype with the co-circulation of two different lineages detected as early as 2016, along with the circulation of several Asian I genotype clusters since 2008 support this hypothesis. Lao PDR shares a border with five countries where DENV circulation is highly active [1, 43, 49]. Exchanges between Lao PDR and its neighboring countries is increasing constantly especially with regards to commerce [4, 5]. In this context, the identification of potential DENV introduction routes into Lao PDR and their impact on dengue epidemiology in the country will be challenging in the future.

Here, GSP demonstrated rapid preliminary information on DENV-2 polymorphisms that were in most cases confirmed by full length envelope sequencing. Tree topology differences could be due to the limited number of nucleotides used for GSP (552nt *versus* 1,485nt for full length envelope). However, GSP analytic sensitivity seemed to be higher compared to full length envelope sequencing. In this study, samples with high Cq (above 30) could be only sequenced using GSP, which can help the investigation of samples with volume limitations or for whom viral culture is not possible.

Identification of DENV serotype(s)/genotype(s) during an outbreak can help improve dengue prevention, especially in endemic countries where the disease severity is still challenging [53]. Improvement of rapid RT-PCR diagnostic tool already help the identification of DENV serotype [5, 15].

As previously described, DENV genotype switch or co-circulation could impact DENV emergence and spread as well as modify its epidemiology profile by increasing the number of infected patients and fatal cases [6–8]. DENV genotypes may display differences in their fitness and virulence. Some studies already suggested a possible link between a sudden switch of DENV genotypes and an increase of disease severity for DENV-1, DENV-2 and DENV-3 in Asia or South America [54–57]. In our series, the four DENV-2 isolates from fatal cases in 2018 and 2019 belonged to the Cosmopolitan genotype/Lineage B which was detected in 2017, but has not had an increase in cases by the end of 2018. Parallel studies are needed to investigate the respective roles of virus pathogenicity and indirect factors such as socio-economic factors governing access to care to determine the real impact of the emergence of DENV genotypes in endemic countries like in Lao PDR [58].

In Lao PDR, herd immunity against the different dengue virus serotypes remains cryptic and it can be assumed to be low for DENV-2, as seen by the background circulation and the various introductions seen throughout this study period.

Virologic investigations are crucial for DENV epidemic prevention and follow up. Direct diagnosis by RT-PCR is at present the gold standard for confirming dengue virus infection during the acute phase of the disease. When the geographic coverage is sufficient across the country, the serotype determination by real time RT-PCR can be used to estimate the proportions of each DENV serotype and generate data that could be used as a proxy to estimate the herd immunity against each. Molecular epidemiology by partial or complete envelope gene sequencing has been used for decades to better understand DENV circulation at regional or inter-continental levels. Genotype determination reinforces the capacity to identify trans-border routes of circulation and establish a more precise timepoint of emergence of a specific cluster of DENV isolates. From that perspective, GSP is a valuable frontline molecular epidemiological tool for countries with limited laboratory resources that are confronted with dengue. Moreover, GSP could be useful to follow the circulation of DENV-2 on both a country and regional scale.

## Supporting information

S1 Table References of DENV-2 envelope gene sequences from GenBank used in this study. (DOCX)
