# Supplementary material for: Rapid genotyping protocol to improve dengue virus serotype 2 survey in Lao PDR
Source: PLoS One. 2020 Aug 7;15(8):e0237384. doi: 10.1371/journal.pone.0237384 (PMC7413503; doi:10.1371/journal.pone.0237384)
Supplement: S1 Table — (DOCX) [file pone.0237384.s001.docx]

**S1 Table. References of DENV-2 envelope gene sequences from GenBank used in this study.**

| **Country** | **Year** | **GenBank accession number** | **DENV-2 genotype** | **References** |
| --- | --- | --- | --- | --- |
| **American Samoa** | 1972 | HM582107 | American | Stell *et al*, 2010 (17) |
| **Columbia** | 1986 | GQ868592 | American | Unpublished |
| **Fiji** | 1971 | HM582099 | American | Stell *et al*, 2010 (17) |
| **French Polynesia** | 1973 | HM582110 | American | Stell *et al*, 2010 (17) |
| **Mexico** | 1992 | AF100469 | American | Leitmeyer *et al*, 1999 (18) |
| **New Caledonia** | 1971 | HM582102 | American | Stell *et al*, 2010 (17) |
| **Puerto Rico** | 1977 | EU056812 | American | Vasilakis *et al*, 2008 (19) |
| **China** | 2014 | KY038915 | Asian I | Unpublished |
| **LaoPDR-Luang Namtha** | 2008 | KY849752 | Asian I | Castonguay-Vanier *et al*, 2018 (4) |
| **LaoPDR-Luang Namtha** | 2009 | KY849757 | Asian I | Castonguay-Vanier *et al*, 2018 (4) |
| **LaoPDR-Luang Namtha** | 2009 | KY849760 | Asian I | Castonguay-Vanier *et al*, 2018 (4) |
| **LaoPDR-Saravane** | 2009 | KY849758 | Asian I | Castonguay-Vanier *et al*, 2018 (4) |
| **LaoPDR-Saravane** | 2010 | KY849755 | Asian I | Castonguay-Vanier *et al*, 2018 (4) |
| **LaoPDR-Vientiane Capital** | 2009 | KY849765 | Asian I | Castonguay-Vanier *et al*, 2018 (4) |
| **LaoPDR-Vientiane Capital** | 2010 | KY849763 | Asian I | Castonguay-Vanier *et al*, 2018 (4) |
| **LaoPDR-Vientiane Capital** | 2010 | KY849767 | Asian I | Castonguay-Vanier *et al*, 2018 (4) |
| **LaoPDR-Vientiane Capital** | 2010 | KY849768 | Asian I | Castonguay-Vanier *et al*, 2018 (4) |
| **LaoPDR-Vientiane Capital** | 2012 | MN628252 | Asian I | Unpublished |
| **LaoPDR-Vientiane Capital** | 2012 | MN628237 | Asian I | Unpublished |
| **LaoPDR-Vientiane Capital** | 2012 | MN628232 | Asian I | Unpublished |
| **LaoPDR-Vientiane Capital** | 2012 | MN628241 | Asian I | Unpublished |
| **LaoPDR-Vientiane Capital** | 2013 | LC147056 | Asian I | Phommanivong *et al*, 2016 (20) |
| **Myanmar** | 2015 | KX357994 | Asian I | Kyaw *et al*, 2017 (21) |
| **Singapore** | 2011 | JN544398 | Asian I | Unpublished |
| **Taiwan (ex Vietnam)** | 2012 | MG895014 | Asian I | Yang *et al*, 2018 (23) |
| **Thailand** | 2004 | KY586602 | Asian I | Salje *et al*, 2017 (26) |
| **Thailand** | 2006 | JF812111 | Asian I | Unpublished |
| **Thailand** | 2007 | JN568273 | Asian I | Warrilow *et al*, 2012 (22) |
| **Thailand** | 2010 | JF968013 | Asian I | Huang *et al*, 2012 (44) |
| **Thailand** | 2011 | MG564087 | Asian I | Yang *et al*, 2018 (23) |
| **Thailand** | 2015 | MK780871 | Asian I | Hamel *et al*, 2019 (43) |
| **Thailand** | 2015 | MK780872 | Asian I | Hamel *et al*, 2019 (43) |
| **Thailand** | 2016 | LC410184 | Asian I | Phadungsombat *et al*, 2018 (25) |
| **Thailand** | 2016 | MK780874 | Asian I | Hamel *et al*, 2019 (43) |
| **Thailand** | 2016 | MK780877 | Asian I | Hamel *et al*, 2019 (43) |
| **Thailand** | 2016 | MK780879 | Asian I | Hamel *et al*, 2019 (43) |
| **Vietnam** | 2006 | EU482542 | Asian I | Unpublished |
| **Vietnam** | 2010 | JN568282 | Asian I | Warrilow *et al*, 2012 (22) |
| **China** | 1987 | AF204178 | Asian II | Unpublished |
| **China** | 1989 | AF204177 | Asian II | Unpublished |
| **Indonesia** | 1975 | GQ398268 | Asian II | Christenbury *et al*, 2010 (27) |
| **New Guinea** | 1944 | AF038403 | Asian II | Gruenberg *et al*, 1988 (28) |
| **Papua New Guinea** | 2008 | FJ906959 | Asian II | Unpublished |
| **Philippines** | ND | AF295697 | Asian II | Unpublished |
| **Philippines** | 1983 | L10045 | Asian II | Lewis *et al*, 1993 (29) |
| **China** | 1985 | AF119661 | Asian/American | Unpublished |
| **Dominican Republic** | 2001 | AB122022 | Asian/American | Anzai *et al*, 2004 (30) |
| **French Guiana** | 2006 | EU920848 | Asian/American | Unpublished |
| **Guadeloupe** | 2006 | EU920850 | Asian/American | Unpublished |
| **Guatemala** | 2009 | HQ999999 | Asian/American | Añez et al, 2011 (31) |
| **Nicaragua** | 1999 | GQ199895 | Asian/American | Unpublished |
| **Nicaragua** | 2007 | GQ199868 | Asian/American | Unpublished |
| **Peru** | 2010 | KC294223 | Asian/American | Williams *et al*, 2014 (32) |
| **Peru** | 2010 | KC294221 | Asian/American | Williams *et al*, 2014 (32) |
| **Puerto Rico** | 1994 | GQ398313 | Asian/American | Christenbury *et al*, 2010 (27) |
| **Puerto Rico** | 2005 | EU687216 | Asian/American | Unpublished |
| **Venezuela** | 1990 | GQ868540 | Asian/American | Unpublished |
| **Vietnam** | 2003 | FM210219 | Asian/American | Unpublished |
| **Vietnam** | 2003 | FM210221 | Asian/American | Unpublished |
| **Vietnam** | 2003 | FM210209 | Asian/American | Unpublished |
| **Bangladesh** | 2009 | JN036373 | Cosmopolitan | Unpublished |
| **China** | 2000 | AF276619 | Cosmopolitan | Unpublished |
| **China** | 2003 | FJ196853 | Cosmopolitan | Wu *et al*, 2011 (33) |
| **China** | 2014 | KP723478 | Cosmopolitan | Zhao *et al*, 2016 (34) |
| **China** | 2015 | KY038897 | Cosmopolitan | Unpublished |
| **China (ex India)** | 2013 | KF479233 | Cosmopolitan | Ma *et al*, 2014 (35) |
| **India** | 2010 | JN568259 | Cosmopolitan | Warrilow *et al*, 2012 (22) |
| **India** | 2011 | JQ955624 | Cosmopolitan | Dash *et al*, 2013 (36) |
| **Indonesia** | 1976 | GQ398264 | Cosmopolitan | Christenbury *et al*, 2010 (27) |
| **Malaysia** | 2013 | KJ806895 | Cosmopolitan | Ng et al, 2015 (37) |
| **Malaysia** | 2014 | KT806325 | Cosmopolitan | Moore *et al*, 2017 (38) |
| **Pakistan** | 2013 | KJ010186 | Cosmopolitan | Akram *et al*, 2015 (39) |
| **Singapore** | 2007 | GQ398267 | Cosmopolitan | Christenbury *et al*, 2010 (27) |
| **Singapore** | 2009 | JF327392 | Cosmopolitan | Grant *et al*, 2011 (40) |
| **Singapore** | 2014 | KX224268 | Cosmopolitan | Hapuarachchi *et al*, 2016 (41) |
| **Sri Lanka** | 1996 | FJ882602 | Cosmopolitan | Unpublished |
| **Sri Lanka** | 2016 | KY495803 | Cosmopolitan | Moore *et al*, 2017 (38) |
| **Taiwan** | 2002 | DQ645546 | Cosmopolitan | Chen *et al*, 2008 (42) |
| **Thailand** | 2015 | MK780863 | Cosmopolitan | Hamel *et al*, 2019 (43) |
| **Thailand** | 2015 | MK780864 | Cosmopolitan | Hamel *et al*, 2019 (43) |
| **Thailand** | 2016 | LC410190 | Cosmopolitan | Phadungsombat *et al*, 2018 (25) |
| **Thailand** | 2016 | MK780867 | Cosmopolitan | Hamel *et al*, 2019 (43) |
| **Thailand** | 2016 | MK780868 | Cosmopolitan | Hamel *et al*, 2019 (43) |
| **Vietnam** | 2006 | EU482672 | Cosmopolitan | Unpublished |
| **Guinea** | 1981 | EF105378 | Sylvatic | Vasilakis *et al*, 2007 (24) |
| **Malaysia** | 1970 | EF105379 | Sylvatic | Vasilakis *et al*, 2007 (24) |
